# The potential for stabilizing Amundsen Sea glaciers via underwater curtains

**DOI:** 10.1093/pnasnexus/pgad103

**Published:** 2023-03-27

**Authors:** Michael Wolovick, John Moore, Bowie Keefer

**Affiliations:** College of Global Change and Earth Systems Science, Beijing Normal University, 19 Xinjiekouwai St, Haidian District, Beijing 100875, China; Glaciology Section, Alfred-Wegener-Institut Helmholtz-Zentrum für Polar- und Meeresforschung, Bremerhaven, Germany; College of Global Change and Earth Systems Science, Beijing Normal University, 19 Xinjiekouwai St, Haidian District, Beijing 100875, China; Arctic Center, University of Lapland, Pohjoisranta 4, 96200 Rovaniemi, Finland; Adjunct, Clean Energy Research Centre, University of British Columbia, 2329 West Mall, Vancouver BC V6T 1Z4, Canada

## Abstract

Rapid sea level rise due to an ice sheet collapse has the potential to be extremely damaging the coastal communities and infrastructure. Blocking deep warm water with thin flexible buoyant underwater curtains may reduce melting of buttressing ice shelves and thereby slow the rate of sea level rise. Here, we use new multibeam bathymetric datasets, combined with a cost–benefit model, to evaluate potential curtain routes in the Amundsen Sea. We organize potential curtain routes along a “difficulty ladder” representing an implementation pathway that might be followed as technological capabilities improve. The first curtain blocks a single narrow (5 km) submarine choke point that represents the primary warm water inflow route towards western Thwaites Glacier, the most vulnerable part of the most vulnerable glacier in Antarctica. Later curtains cross larger and deeper swaths of seabed, thus increasing their cost, while also protecting more of the ice sheet, increasing their benefit. In our simple cost–benefit analysis, all of the curtain routes achieve their peak value at target blocking depths between 500 and 550 m. The favorable cost–benefit ratios of these curtain routes, along with the trans-generational and societal equity of preserving the ice sheets near their present state, argue for increased research into buoyant curtains as a means of ice sheet preservation, including high-resolution fluid-structural and oceanographic modeling of deep water flow over and through the curtains, and coupled ice-ocean modeling of the dynamic response of the ice sheet.

**Significance**

Collapse of the West Antarctic ice sheet is a major risk, with ocean melting having already destabilized the equivalent of a meter of global sea rise, and the Thwaites ice shelf recognized as the most vulnerable sector of the ice sheet. Reducing greenhouse gas emissions cannot stabilize the ice sheet, which needs to re-establish contact and regain buttressing on sea floor highs. We explore siting options for subsea curtain barriers that might be able to block deep warm ocean currents and thus protect Thwaites and other nearby ice shelves. Our preliminary cost–benefit analysis helps to clarify societal options in the event of rapid ice sheet retreat and motivates future work on the glaciological, oceanographic, engineering, ecological, and governance questions raised by these barriers.

Ice sheet collapse has long been one of the most feared consequences of climate change, particularly for the West Antarctic ice sheet (WAIS, e.g. (1)). This fear has largely been driven by a growing understanding of the marine ice sheet instability (MISI), the dynamic instability created by a marine-based ice sheet whose bed deepens inland (2–5). The onset and the rate of MISI collapse are strongly dependent on ocean thermal forcing (TF), as warm salty waters at depth cause high basal melt rates underneath floating ice shelves, thereby thinning the shelves and reducing the stabilizing buttressing force that they provide (references therein (6)). In the Amundsen Sea sector of WAIS, this ocean TF is provided by modified circumpolar deep water (CDW), which has triggered high basal melt rates underneath floating ice shelves (e.g. (7)), and these high melt rates have in turn led to grounding line retreat and mass loss of the grounded ice sheet (8). Because the grounding line at multiple important glaciers in the Amundsen sector is currently retreating down a retrograde slope, there have been plausible suggestions in the literature that the present-day WAIS retreat represents the beginning stages of a MISI collapse (9–11).

Preliminary research into targeted glacial geoengineering (2–6) aims to design an intervention, at the scale of a large but achievable civil engineering project, with the potential to prevent, delay, or at least slow down a marine ice sheet collapse. Wolovick and Moore (17) proposed using solid artificial sills to block warm water from reaching the grounding line; using a simple model of ice flow and basal melting, they found that reductions in the basal melt rate could cause the floating ice shelf to thicken and flow outward, ultimately regrounding on the sill, which increased buttressing, reduced ice flux across the grounding line, and stabilized the ice sheet. In a companion paper (18), we improve on the (17) design by introducing the idea of buoyant underwater curtains. These are flexible impermeable curtains anchored to the seabed and held upright in the water column by their own buoyancy. These could be constructed and installed in a temperate ocean environment using present-day ocean construction and logistics capabilities. Installation in a polar ocean would be a logistical challenge, but still potentially achievable for much lower cost than traditional coastal protection, which is estimated to be 20–70 billion USD per year (19, 20). While it is true that even a perfect ice sheet intervention would not completely remove the need for coastal protection, since thermal expansion, the melting of smaller glaciers and ice caps, and ablation of the Greenland ice sheet will still produce sea level rise in a warming climate, none of these other sources have the potential to raise sea level at the extreme rates and magnitudes that could be realized from the tail risk of a rapid marine ice sheet collapse (21). In addition, the principle of underwater curtains has been demonstrated at scale by “temperature control curtains” that are used to moderate the outflow temperatures from stratified hydroelectric reservoirs (22–24). Buoyant curtains have a number of advantages over the (17) sills, including lower costs, greater resilience to iceberg impacts, less impact on local marine ecosystems during installation, and ease of removal in the event of unforeseen side effects.

To be clear, we do not advocate for deployment of ice sheet interventions in either the short or the medium term. There are many scientific, engineering, and political unknowns that need to be addressed before even small pilot projects could begin, much less large-scale deployment in the Amundsen Sea. What we aim to do here and in Keefer et al. (in press) is to develop an agenda for future research, organized around a schematic proposal with many intermediate steps along the way. Here we hope to make a small contribution forwards using newly released multibeam bathymetry data (24) to quantitatively evaluate different potential barrier routes in the Amundsen Sea (Fig. 1). We develop a multi-part cost metric that can be used to evaluate the relative difficulty of different curtain routes based on their bathymetric profile and the desired blocking depth. We compare this cost metric with a benefit metric based on the change in TF at the blocking depth to determine the optimal blocking depth for each curtain location. Our findings about which possible barrier routes show the most potential can provide a starting point for future studies with more advanced ocean or ice-ocean models.

**Fig. 1.**
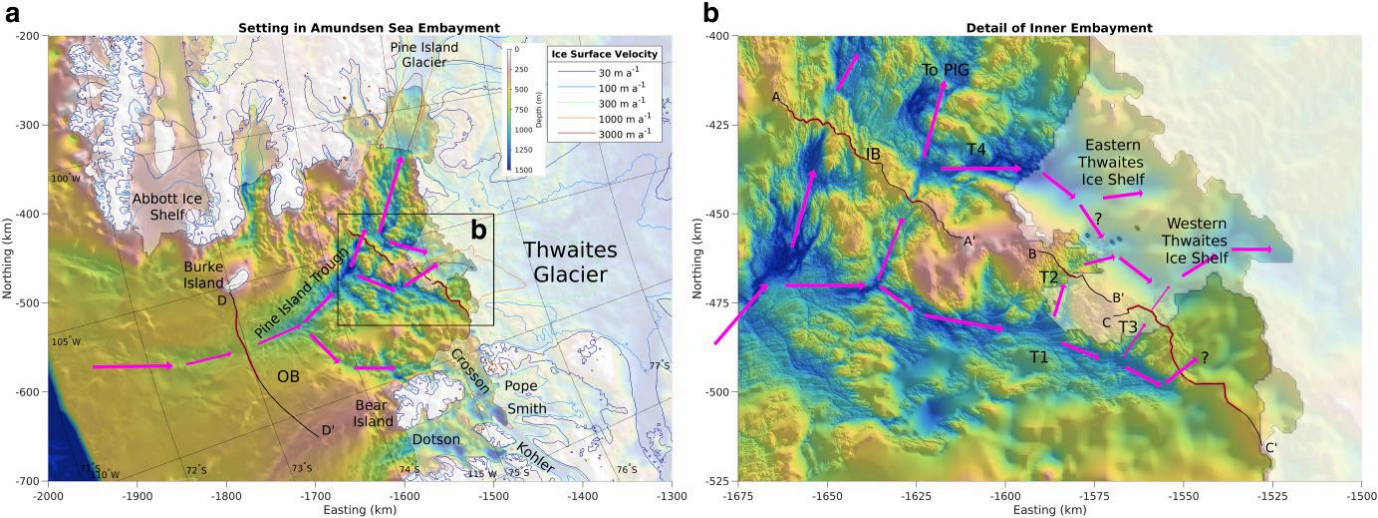
Physical setting in the Amundsen Sea Embayment. a) Hillshaded bathymetry from BedMachine Antarctica (25). Shaded regions indicate grounded and floating ice; colored lines show ice surface velocity contours from MEaSUREs version 2 (26, 27). Thin black lines show the full length of candidate curtain routes; thick red lines show the segments of those routes below the best-value blocking depth. b) As in (a), but detailed bathymetry taken from Ref. (24) at 50 m resolution. Trough numbering scheme T1–T4 follows (24). IB, inner bay curtain route; OB, outer bay curtain route. Arrows indicate approximate route of warm CDW from the continental shelf break through the trough system around and under Thwaites.

## Results

**Fig. 2.**
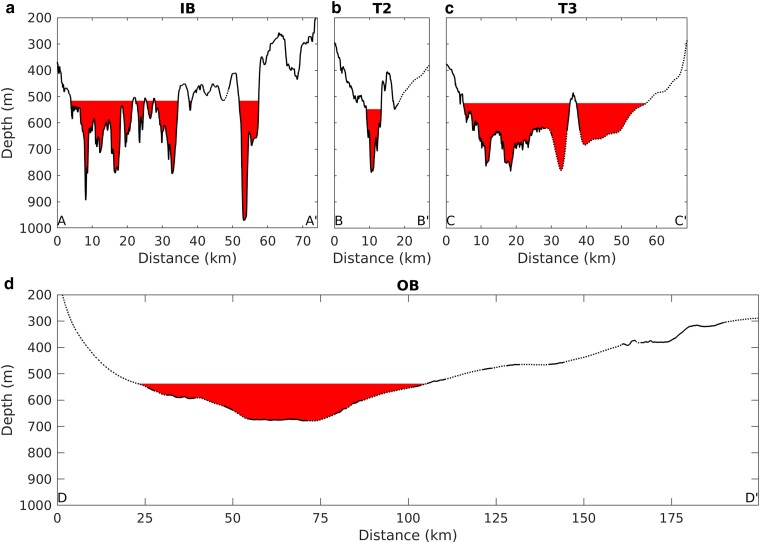
Comparison of bathymetric profiles along four candidate curtain routes. Top row a–c) shows routes IB, trough 2 (T2), and trough 3 (T3), which together form a proximal route protecting both PIG and Thwaites (Fig. 1). Bottom row d) shows the OB route, which protects those two glaciers plus Crosson ice shelf. Solid lines indicate where the profile is constrained by nearby multibeam echo-sounding data, dashed lines indicate where the profile relies on interpolation or gravity inversions. Capital letters (A, A′, etc.) indicate start and end points of profiles in Fig. 1. Vertical exaggeration for all profiles is 75.

We choose four candidate routes to investigate, three of which can be grouped into a combined proximal route, and one of which is a standalone distal route (Fig. 1). The distal route, which we term the outer bay (OB) route, cuts across the Amundsen Sea embayment from Burke Island to Bear Island, crossing Pine Island Trough at its shallowest point (Figs. 1a and 2d). The OB curtain route blocks warm CDW from reaching all of the major glaciers in the Amundsen Sea, including Pine Island Glacier (PIG), Thwaites, and the smaller Pope, Smith, and Kohler Glaciers which feed into the Crosson ice shelf. The proximal combined route (Fig. 1b) is composed of a route crossing inner Pine Island Bay (inner bay (IB), Fig. 2a), plus routes crossing Thwaites troughs 2 and 3 ((T2 and T3), Fig. 2b and c; we follow (25) for the numbering of troughs near Thwaites Glacier). Wahlin et al. (29) found that T2 was the primary pathway through which ocean heat entered the cavity underneath the western Thwaites ice shelf, while T3 was a secondary pathway. The IB route prevents deep warm water from flowing towards both PIG and the eastern Thwaites ice shelf. We also considered curtain routes crossing T4 to protect eastern Thwaites alone, but given that T4 is much wider and deeper than the troughs in the IB route, and given that a potential T4 curtain would protect a smaller area of the ice sheet than the IB route, we found that the T4 curtain routes are unequivocally lower value than the IB route, and we do not consider them further.

**Fig. 3.**
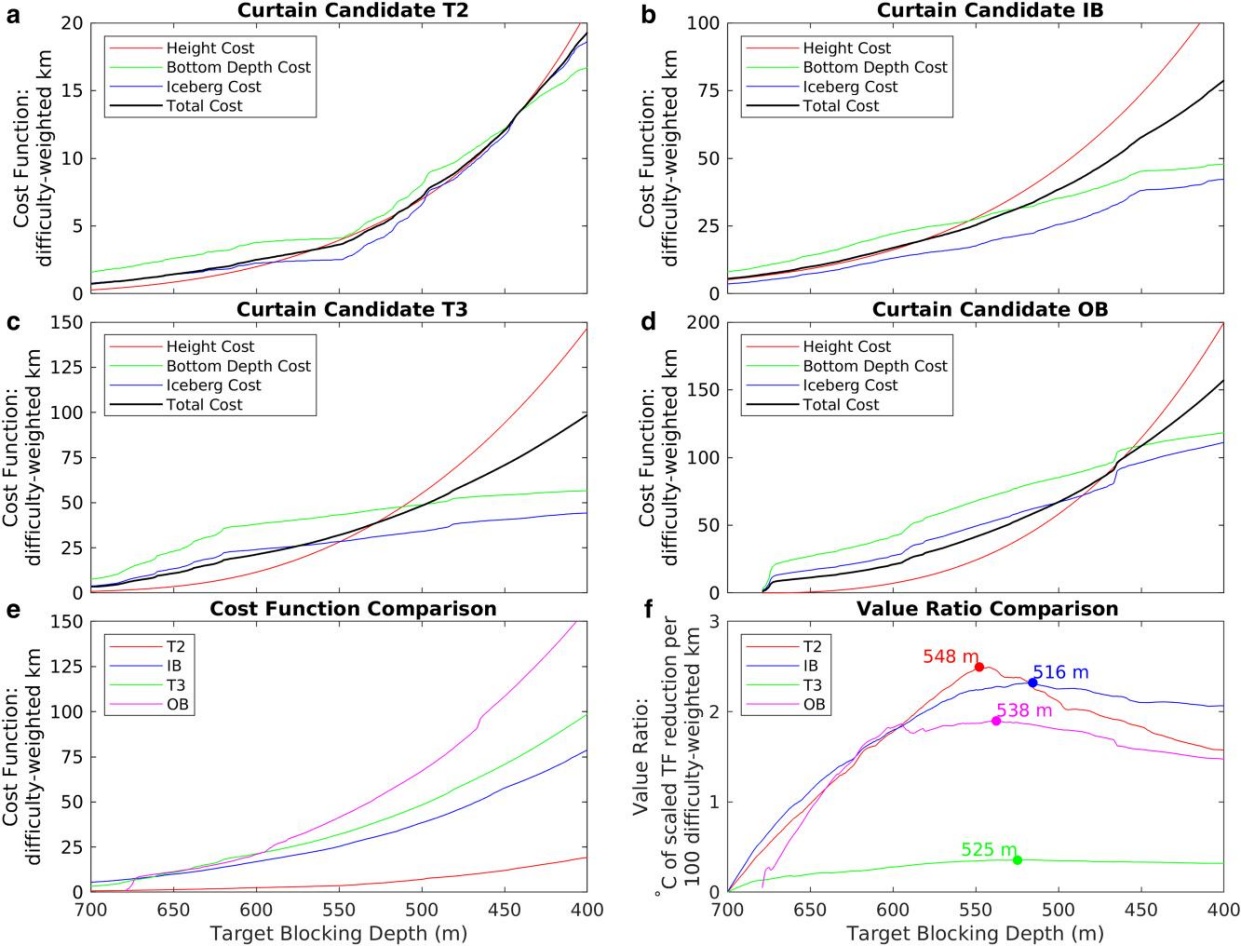
Comparison of cost functions and value ratios for all four curtain candidates. a–d) Cost function components for each of the four candidates as a function of target blocking depth. Note change in *y*-axis scale between each plot. e) Comparison of total cost function between the four routes as a function of target blocking depth. f) Comparison of value ratio for the four routes as a function of blocking depth. Note that the TF reduction for each route has been scaled by the VAF protected by that route as described in the text. Best-value blocking depths are labeled for each route.

Within the proximal routes, T2 has the best value and the lowest cost. This route is characterized by a single narrow trough less than 800 m deep and 4–5 km wide (Fig. 2b). The trough is well constrained by multibeam data, but above about 500–550 m, the flanks of the trough spread out into more gradual slopes and the bathymetry on one side is unreliable. The cost function components for T2 are all below 5 difficulty-weighted km for blocking depths below 550 m, after which they increase sharply (Fig. 3a). This increase in cost for shallower depths results in a sharply peaked value ratio, with a well-defined maximum in value occurring at a blocking depth of 548 m (Fig. 3f). At this depth, only 4.3 km of curtain length is required, with an average height above the seabed of 130 m (Table 1).

**Table 1.** Summary of route statistics.

Route	Best blocking depth (m)	Mean sea depth (m)	Max sea depth (m)	Mean height (m)	Max height (m)	Length (km)	Cost fctn (km)	Data quality (% multibeam)	Regions protected
T2	548	678	788	130	240	4.3	3.7	100	W. Thwaites (primary CDW pathway)
IB	516	643	971	127	455	35	34	100	PIG and E. Thwaites
T3	525	645	783	121	258	50	40	53	W. Thwaites (secondary CDW pathway)
OB	538	617	679	79	142	82	47	46	PIG, all Thwaites, and Crosson ice shelf

*Notes*: This table summarizes the statistics for the four candidate curtain routes. All statistics are evaluated for the best-value blocking depth. Data quality refers to the fraction of bathymetric data along the route that is based on high-quality multibeam data, as opposed to gravity inversions or interpolation.

The next best value of the proximal routes is IB. The IB route crosses multiple narrow troughs nearly 1000 m deep (Fig. 2b). In between the troughs, route IB follows seabed ridges that are generally 500–600 m deep. The overwhelming majority of the IB route is constrained by multibeam data. The cost function for IB is generally about 5–7× the value of the cost function for T2, but because the benefit function for IB is weighted about 5× more strongly than for T2 (as IB protects a much larger area of the ice sheet), their value ratios are similar (Fig. 3f). The best-value blocking depth for IB is slightly shallower, at 516 m, and the curtain length required is about an order of magnitude larger, at 35 km (Table 1). The greatest technical challenge to building a curtain along route IB will likely be the deepest trough, which requires a foundation depth of 971 m and a maximum curtain height of 455 m above the seabed, although the mean curtain height along the IB route is only 127 m above the seabed.

The lowest value proximal route is T3, which protects the secondary CDW inflow pathway towards the western half of Thwaites Glacier. Nearly half of the T3 route is reliant on gravity inversions rather than multibeam data for its bathymetric profile. Because T3 protects much less of the ice sheet, its value ratio is much lower, a clear outlier with respect to the other three routes (Fig. 3f).

Finally, the distal OB route runs through glacial sediments deposited further out on the continental shelf, and is therefore characterized by smooth slopes and broad shallow troughs. The maximum depth of OB is less than 700 m (Fig. 2 and Table 1), but the width of the trough at this depth is approximately 20 km. Although large sections of the OB profile are reliant on interpolation between sparse echo soundings, the deepest part of the profile is well characterized by multibeam data, giving us confidence that the smooth, broad, and relatively shallow bottom is real. The broad shallow slopes of the OB route ensure that curtain length increases steeply as the target blocking depth becomes shallower, resulting in the highest values of the cost function of any of the four routes (Fig. 3d and e). However, because the OB route also protects more of the ice sheet than the other routes, its value ratio is only slightly lower than the much smaller T2 and IB routes (Fig. 3f). Because of the broad smooth nature of the seabed on the continental shelf, the OB curtain has the longest length but the smallest height above the seabed of all four of the routes that we considered (Table 1). Note that, while we have assumed a constant blocking depth for our analysis, in reality the thermocline slopes downwards from east to west across the continental shelf (30), so a future design refinement may incorporate a variable blocking depth for this route.

## Discussion

It would be prudent to have a gradual implementation sequence, such that easier, and more proportionately high-impact, curtains are installed before larger ones. The proximal route offers such a sequence. Available evidence suggests that trough T2 is the primary entrance route for CDW approaching the western Thwaites grounding line (29), and this trough is barely 5 km wide, creating a favorable ratio of impact to cost. Once humanity gained experience at T2, further curtains could be constructed across multiple individual troughs along the IB route, thus blocking CDW transport towards PIG and the eastern half of Thwaites. Finally, if further intervention was still needed, a curtain could be built to block warm water transport through T3, thus sealing the last entrance towards western Thwaites.

The distal route, by contrast, does not offer a gradual implementation sequence. However, installation on hard bedrock is more challenging than on soft sediments because of the need for rock drilling and shaping, the need to accommodate greater bed slopes, and sharper gradients in curtain shape and height, resulting in more complex deformation modes (Keefer et al., in press). The smoother bathymetry of the distal route, by contrast, allows for a curtain that is consistently lower to the seafloor, with a maximum height only slightly larger than the mean height (Table 1). Thus, we have reason to believe that the real cost of the distal route may be lower than that of the proximal route at equal values of the cost function.

The biggest research priorities needed to evaluate the potential of curtains as a glaciological intervention are (1) high-resolution fluid-structural modeling and tank tests to determine the “leakiness” of the curtain design and (2) coupled ice-ocean modeling to determine circulation changes, melt changes, and ice-dynamic response as a result of curtain installation.

The potential for “leakiness” of the curtains is one of the largest unknowns impacting their effectiveness. We envision that the curtains would be composed of separate overlapping panels in order to allow them to deform around impinging icebergs and minimize damage (Keefer et al., in press). This design will produce gaps between adjacent panels, through which some water will leak, especially if the curtains experience fluid-structural oscillations. In addition, the literature on bathymetric sills suggests that even a gapless curtain would not be expected to completely block the transport of deep warm water. Water masses can be drawn up from depth and transported over bathymetric sills even when the sill crest is shallower than the origin of the water mass (e.g. (32, 33)). In addition, mixing between the blocked layer and overlying water masses can entrain deep water and carry it over the sill (e.g. (34)). However, the transport of deep water over the sill is still less than it would be for an unobstructed flow (35, 36) which is consistent with glaciological evidence that glaciers protected by shallow sills are less sensitive to ocean forcing than glaciers without such protection (36). Either high-resolution modeling capable of resolving both deformation modes of the curtain and turbulence in the surrounding ocean or scale model tank tests are needed to quantify the actual ocean heat flux reduction that would be caused by the curtains.

In addition, research is also needed on the larger-scale circulation changes curtains might cause, and on whether those changes would help or hinder the effectiveness of the curtains. For example, Gurses et al. (37) used an ocean circulation model to investigate the impact of a very large wall built to cut off the entire Amundsen Sea Embayment. They found that while melt rates at the protected glaciers were reduced, some of the blocked warm water was rerouted to other ice shelves, reducing the net benefit of the wall. While the enormous wall they considered in their model dwarfs the curtains we propose, the general cautionary point they raise is an important one. For instance, while T2 may be the primary ingress route for warm CDW towards western Thwaites at the present day, we cannot assume that it will remain so if it were blocked by a curtain. Blocking warm CDW from entering at T2 could potentially cause the water to reroute and enter western Thwaites through T3 or IB, which would be much more costly to block. As the bathymetry under the ice shelf is largely unknown, further research will be needed to investigate the possibility of deep connections between eastern and western Thwaites.

Finally, some have objected to research into targeted geoengineering because “the limited resources available should instead be used to address the root causes of accelerating ice loss—namely emissions and human-induced climate change” (38). This reflects the longstanding concern about the potential for moral hazard with geoengineering research (39). However, there is a very real risk that humanity may be faced with a MISI collapse even in low-emissions scenarios. Recent work has suggested that the observed increase in upwelling-favorable winds in the Amundsen Sea (which is the proximal cause of increased CDW transport towards the grounding line and increased melt rates) can be attributed roughly evenly between a forced greenhouse gas response and internal climate variability (40). Internal variability in the ocean forcing affecting an ice sheet that is subject to MISI produces a broad and skewed probability distribution for future sea level rise, with substantial tail risks of very rapid ice sheet collapse even in scenarios without mean warming (41). Thus, all emissions scenarios contain a tail risk of very rapid MISI collapse. While that risk may be higher in warmer scenarios, we cannot rule out the possibility that humanity may be faced with a dangerously rapid ice sheet collapse in the coming decades or centuries even after we have cut emissions aggressively.

There is therefore a strong argument to be made in favor of preserving the ice sheets as close to their present-day configuration as possible, on the basis of both socioeconomic and intergenerational equity. The alternative to ice sheet preservation is not emissions reductions; rather, in the event that the tail risk of rapid ice sheet collapse is realized, the alternative to ice sheet preservation will be widespread spending on local coastal protection by nations and communities that can afford it, and retreat from the coast for those who cannot. Stopping, or at least slowing, the sea level rise at the source is a harm-reduction measure that benefits vulnerable communities nearly equally. Any construction in the Amundsen Sea would require detailed impact assessments to be done under the framework of the Antarctic Treaty System and especially the Madrid Protocol. The potential impact of curtains on marine life needs to be thoroughly researched before any intervention could begin. While preliminary research can be undertaken within the existing limits of the Treaty, deployment will probably require unanimity amongst the parties, which is a high bar to clear (42). Nonetheless, ice sheet preservation may be attractive to Treaty parties, as these nations must justify their exclusive control over the Antarctic to the broader international community and they therefore have an incentive to be seen acting for the common good (43). In addition, an argument could be made that there is actually a duty to act to preserve the ice sheets under the precautionary principle, since an ice sheet collapse will be an effectively irreversible change (44). Should we allow the ice sheet to collapse, the result would be massive changes both to the local environment and to coastal societies and ecosystems worldwide. By contrast, preserving the ice sheet at roughly its present state preserves the freedom of action of future generations.

## Conclusions

The latest Intergovernmental Panel on Climate Change (IPCC) report used the calibrated language *limited evidence* and *low confidence* to describe the possibility that various forms of geoengineering, including localized interventions, might reduce the sea level commitment from MISI (21). We agree with these caveats, and we emphasize that humanity is a very long way from being able to implement any sort of targeted glacial geoengineering. Nonetheless, there is a very real risk that we may be faced with an uncontrolled ice sheet collapse at some point in the coming decades or centuries. If we do find ourselves in one of those unfortunate timelines, it would be better if we are prepared with well-considered contingency plans, rather than rushing to implement a half-baked idea that has not received careful scrutiny. As suggested in Ref. (17), it will take many rounds of design iteration and extensive research before the scientific and engineering communities produce something approaching an implementable design.

Our results suggest that trough T2 may be the highest-leverage location for an intervention, where the primary ingress route for CDW into the Thwaites sub-ice cavity may be blocked at 550 m depth with a curtain only 4 km long, and a cost function an order of magnitude smaller than any of the other routes we considered. However, this conclusion depends on the assumption that blocking T2 will not cause water to reroute through T3; if that assumption is violated, then T2 could be combined with IB and T3 to produce a combined proximal route that protects all of PIG and Thwaites, albeit for substantially higher cost. The longer OB route has an easier seabed setting than the proximal route and protects Crosson ice shelf as well. In our simplified cost–benefit analysis, all of the candidate routes reach peak value at target blocking depths of 500–550 m. This finding can serve as a starting point for future experiments with ocean circulation and ice flow models, but it should not be viewed as the final word on blocking depth.

Indeed, the primary purpose of this and our companion paper is not to propose a finished design, but rather to make a plausible schematic proposal that can then be the focus of future research. Both fluid-structural modeling and scale model tank tests are needed to quantify mixing of warm water through and over the curtains and to understand curtain deformation during iceberg encounters. Mixing parameterizations derived from these experiments can be used in ocean model experiments to quantify how different blocking heights and curtain routes actually translate into changes in ice shelf basal melt rates. Dynamic ocean models will also be able to evaluate the extent to which transient upward excursions in the thermocline may overtop the curtain, which could potentially push the best-value blocking depths to shallower (and thus more expensive) levels than those calculated with our benefit function based on the mean oceanographic profile. Changes in melt rates can be used as forcing for ice flow models to determine whether an intervention would indeed produce societally beneficial changes in the magnitude, rate, or timing of sea level rise. Coupled ocean circulation and ice flow modeling is needed to produce more robust estimates of the response of the ice-ocean system. Attention is needed from marine biologists to quantify the likely side effects of curtains on local marine ecosystems, both during the initial installation and in the long term. Before any intervention could be attempted in the Amundsen Sea, smaller pilot projects would need to be constructed at more accessible locations elsewhere in the world to demonstrate feasibility, verify models, and of course, to identify unanticipated consequences that may have gone unnoticed in earlier research. Last, but not least, work is needed from political and social scientists to understand how the peoples of the world view deliberate interventions in the ice sheet system, and to devise legitimate decision-making structures that can balance the needs of many different stakeholders within a context of humanity’s collective need for a stable sea level.

## Materials and methods

Keefer et al. (in press) estimate the cost of 80 km long curtain to be about 40–80 billion USD spread over a decade of construction, followed by 1–2 billion per year in maintenance. Costs estimated for deployment of stratospheric aerosol injection are put at 7–70 billion USD/year (44), while carbon capture and storage costs are around 20–300 USD/ton with perhaps 8–15 Gt/year of removal needed to achieve a 1.5C future climate pathway (45), figures which imply costs of 160–4500 billion USD/year. Thus, costs of the most commonly considered geoengineering solutions are uncertain by an order of magnitude, and targeted glacial interventions are competitive with these planetary-scale interventions, although these larger-scale interventions address many harmful impacts of climate change beyond simply rising sea levels.

We manually pick candidate curtain routes on the bathymetric map (Fig. 1), guided by the principle of following ridges where possible and only crossing troughs where they are narrowest, shallowest, or both. We interpolate the bathymetry onto each route from a high-resolution multibeam bathymetric compilation (25), supplemented by (26) in data gaps and areas not covered by the high-resolution map. A critical unknown in our analysis is the target blocking depth; we define the target blocking depth to mean that a curtain must fill the entire space from the seabed up to that depth along its entire route. We do not predict changes in deep ocean circulation in response to the installation of curtains in this paper, so we make the conservative assumption that all areas of the route below the target depth must be blocked in order for the curtain to be effective. Shallower blocking depths thus require both longer and taller curtains, increasing costs, while also producing greater reductions in TF at the grounding line, increasing benefits. We quantify these two factors with a cost function and a benefit function, and we use the ratio between them to identify the best-value blocking depth for each route.

The two fundamental assumptions underlying our method are (1) the dominant factor controlling variance in curtain cost between different routes in the Amundsen Sea will be the bathymetry, which in many cases is known; and (2) the dominant factor controlling curtain effectiveness will be the height of the curtain top within the thermocline, which is also known. Factors that make work in the Amundsen Sea very difficult, such as transportation across rough seas from Punta Arenas, the need for hardening against sea ice, and work stoppages due to iceberg obstruction, are factors shared between all potential routes; the variation between routes should be due to site-specific factors, of which bathymetry is the most important. Thus, by comparing the known oceanography with the known bathymetry, and correcting for the fact that different curtain routes protect different ice sheet catchments, we can estimate the relative value of different curtain routes and thus provide a starting point for future research.

### Cost function

Our multi-part cost function reflects the major factors that increase the degree of difficulty in construction and maintenance of underwater curtains. Those factors are (1) curtain height above the seabed, (2) depth of foundation, and (3) likelihood of iceberg impacts to the foundation. Since each component of the cost metric takes the form of an integral along the path of a potential curtain route, we combine them by expressing them all in units of length. The resulting cost function can be regarded as the difficulty weighted length of a particular curtain route for a given blocking depth. Each term has a single free parameter representing a characteristic scaling value of the variable in question.

#### Curtain height

Oceanographic loads on the curtain increase strongly with height above the seabed. Greater loads place greater demands on the entire structure, requiring more tensile strength and greater buoyancy in the curtain panels as well as more robust foundations. Hydrostatic loads scale quadratically with curtain height (Keefer et al., in press), while hydrodynamic loads only scale linearly, so we use a quadratic height term in our cost function:


(1) $$C_{H}\;=\int_{D_{B}(x)>D_{T}}{\left(\frac{H(x)}{H_{0}}\right)}^{2}dx,$$

where *C*_*H*_ is the height component of the cost function, *H*(*x*) = *D*_*B*_(*x*) − *D*_*T*_ is the height of the curtain above the seabed, and *H*_0_ is the characteristic scaling height for static loads. We take *H*_0_ = 150 m; this corresponds to a tensile load in the curtain of approximately 3 × 10^4^ N m^−1^, a density contrast of Δ*ρ* = 0.5 kg m^−3^, and a lean angle of 30° (Keefer et al., companion paper).

#### Foundation depth

Construction in deep water is likely to be harder than construction in shallow water. Therefore, we add a seabed-depth-weighted term to our cost :function,


(2) $$C_{D}\;=\int_{D_{B}(x)>D_{T}}\frac{D_{B}(x)}{D_{0}}\;dx,$$

where *C*_*D*_ is the depth component of the cost function and *D*_0_ is a characteristic scaling depth. We choose *D*_0_ = 700 m, the base of the thermocline, as the purpose is to block deep warm water. Thus, developing the ability to install curtains in waters about as deep as the thermocline is a common cost, the same for all routes. By contrast, routes that cross troughs much deeper than the thermocline will be penalized, as they may require the development of additional advanced installation techniques not needed for shallower routes.

#### Iceberg impacts

Shallower curtains will be more impacted by icebergs than deep curtains. Furthermore, we expect that impacts between iceberg keels and the foundation modules will be more damaging than impacts with the flexible part of the curtains, which are designed to deform around the bergs. We therefore construct a cost function that scales inversely with foundation depth:


(3) $$C_{B}\;=\int_{D_{B}(x)>D_{T}}{\left(\frac{D_{B}(x)}{D_{\mathrm{berg}}}\right)}^{-1}dx,$$

where *D*_berg_ is a characteristic iceberg keel depth. We take *D*_berg_ = 400 m as a representative ice shelf draft. Thwaites’ frontal ice shelf draft is about 300 m, while PIG has a frontal draft of about 500 m (26). Ice shelf draft may decrease between now and the eventual construction date, but if the curtain performs as planned, then the sub-shelf melt rate should be reduced and iceberg draft should be increased; thus, the mean present-day ice shelf draft is a reasonable choice.

#### Overall cost function

The overall cost function is a weighted sum of the individual components:


(4) $$C=0.5C_{H}+0.25C_{D}+0.25C_{B},$$

where *C* is the overall cost function, which has units of (difficulty-weighted) length, and the weights 0.5 and 0.25 have been chosen to provide an illustrative example. We have chosen to emphasize the curtain height term in the overall cost function as increasing oceanographic loads on the curtain, combined with the requirement that the curtain remain roughly upright within the water column, produce cascading increases in the design requirements for every component of the curtain, including tensile strength, lateral reinforcement, buoyancy, and foundation mass; as our design analysis indicated (Keefer et al., in press), curtain height above the seabed is likely to be the single biggest determinant of curtain difficulty.

### Benefit function

**Fig. 4.**
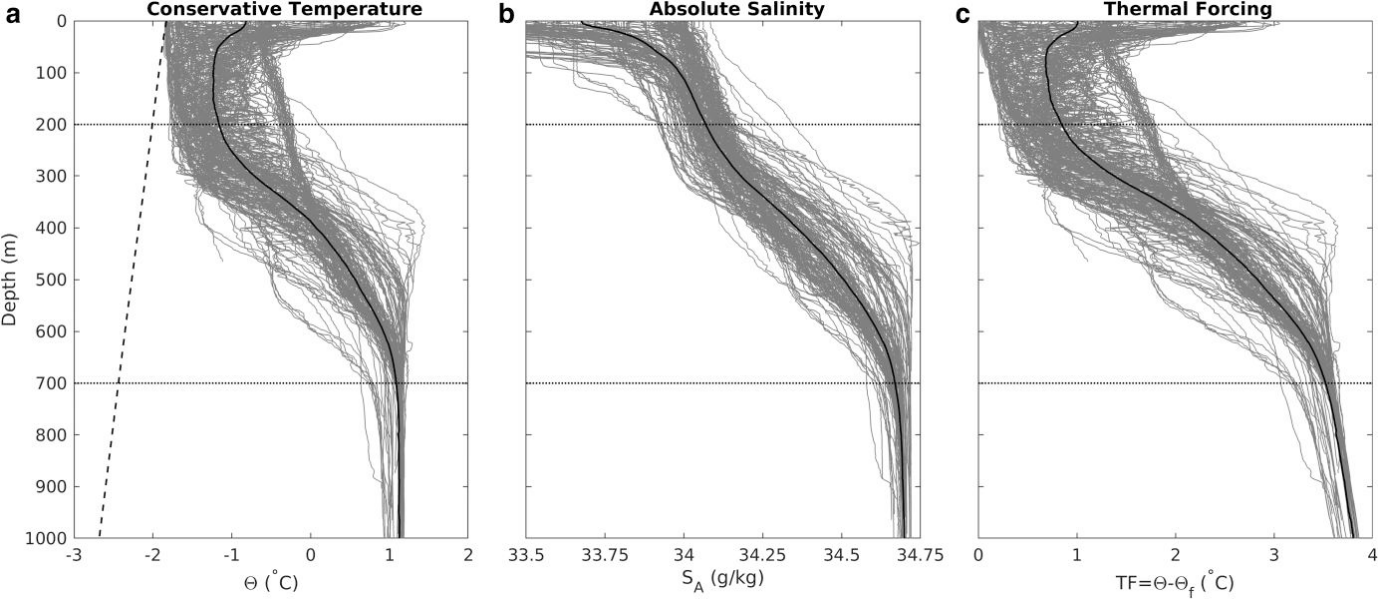
Summary of ocean properties used to define our benefit function. Data were derived by downloading all CTD casts in the World Ocean Database in Pine Island Bay. Thin gray lines are individual casts, thick black lines are mean profiles. a) Conservative temperature (Θ), b) absolute salinity (*S*_*A*_), and c) TF (Θ − Θ _*f*_). Diagonal dashed line in (a) represents the in situ freezing point calculated from the mean salinity and pressure profiles. Horizontal dotted lines in all panels represent the approximate upper and lower limits of the thermocline.

We evaluate potential curtain routes based on comparing the known bathymetry with the known oceanography. We therefore collected all conductivity, temperature, and depth (CTD) casts in the World Ocean Database in the Amundsen Sea, binned them by depth, and computed mean profiles of conservative temperature (Θ) and absolute salinity (*S*) (Fig. 4). We used the mean profiles to compute TF, which is the difference between temperature and the in situ freezing point (TF = Θ − Θ_*f*_). We use the reduction in TF between the bottom of the thermocline, taken to be 700 m, and the curtain top depth as our measure of effectiveness. For the OB curtain route (Fig. 1), the maximum depth along the curtain profile was shallower than 700 m, so for that route we measured TF reduction relative to the maximum depth instead. TF in the Amundsen Sea Embayment is an increasing function of depth for all depths deeper than the ice shelf draft (300–500 m, cf. Fig. 4c), so we assume that the maximum TF capable of reaching the grounding line will be the TF at the blocking depth. Measuring TF at this depth ignores both the fact that some water from below the blocking depth may leak across the curtain, and the fact that the curtain will impose drag on ocean layers some distance above its top. The detailed fluid-structural modeling that would be required to accurately characterize ocean heat flux over and through the curtain is beyond the scope of this paper. We simply assume that the unimpeded flow of water above the blocking depth will be sufficiently large relative to the impeded flow below the blocking depth that the water mass properties glacierward of the curtain will be dominated by the unimpeded flow.

Additionally, different curtains protect different regions of the ice sheet, and some regions are more likely to trigger an unstable collapse than other regions. For example, an intervention that only protected Thwaites would be less likely to prevent an ice sheet collapse than an intervention that protected both PIG and Thwaites. We therefore use the volume above flotation (VAF) of the glaciers protected by each curtain as a proxy for this area-protected effect. We measure VAF totals using the ice thickness map from Ref. (26) and the catchment boundaries from Ref. (46). We set the VAF of Thwaites Glacier to 1 and measure other glaciers relative to this value; under this convention, PIG has a value of 0.79 and the combination of Pope, Smith, and Kohler glaciers has a value of 0.09. For routes that only block part of the approach to Thwaites, we give partial credit under the convention that Western and Eastern Thwaites are both worth 0.5, while the two entrances to Western Thwaites (T2 and T3) are split further to 0.25 each. The weights given to each curtain candidate are thus as follows: T2 = T3 = 0.25 (both given credit for half of Western Thwaites), IB = 1.29 (protecting Eastern Thwaites and all of PIG), and OB = 1.88*R* (protecting PIG, all of Thwaites, and Pope/Smith/Kohler glaciers). Scaling the TF reduction by these values allows us to produce a benefit function that is comparable between different routes, and the ratio of the benefit function over the cost function can be used to select a best-value blocking depth for each curtain route.

While this approach to calculating a benefit function is crude, these weights do capture the general picture of societal risk posed by the different ice-dynamic regions of the Amundsen Sea. A full treatment of this problem would require a coupled ice-ocean model with a large enough ensemble to meaningfully evaluate the change in probability that various societally significant thresholds for sea level rise magnitude, rate, or timing are exceeded when ocean heat flux through key specific choke points is reduced. If a fully coupled model is not available, then melt rates from a standalone ocean model could be used as forcing for a standalone ice sheet model. In either case, the ocean model would either need to be informed by high-resolution fluid-structural modeling that quantified heat flux reduction as a function of curtain height and ambient oceanographic conditions, or else the “leakiness” of the barrier could be treated as a free parameter whose influence could be explored alongside other model parameters. As with any geophysical problem, there is a spectrum of complexity at which the problem of quantifying curtain benefit could be approached. We leave more advanced treatments of the problem for future work. For our purpose, here we aim for the lowest level of complexity that is informed by both the known oceanography and the relative societal importance of different ice sheet sectors.

## Data Availability

Geographical coordinates and bathymetric profiles along the curtain routes, together with statistics and the cost and benefit functions for each route as a function of blocking depth, can be found at https://doi.org/10.5281/zenodo.7746143.
